# Celiac Disease Presenting With Erythema Nodosum in a Child: Case Report and Review of the Literature

**DOI:** 10.1155/crpe/9684135

**Published:** 2026-05-18

**Authors:** Momcilo Pavlovic, Karolina Berenji, Zeljko Rokvic

**Affiliations:** ^1^ Department of Gastroenterology, Children’s Ambulatory Care Center, Subotica, Serbia; ^2^ Department of Hygiene and Human Ecology, Public Health Institute, Subotica, Serbia, phi.org; ^3^ Medical Department, The College of Vocational Studies, Subotica, Serbia

**Keywords:** celiac disease, erythema nodosum, gluten-free diet

## Abstract

Celiac disease (CD) is a chronic autoimmune enteropathy with a wide spectrum of gastrointestinal and extraintestinal manifestations. Erythema nodosum (EN), the most common form of panniculitis in children, has only rarely been reported in association with CD, and the nature of this relationship remains unclear. We describe the case of a 2.5‐year‐old girl who presented with erythematous nodules on the lower extremities, which had been present for 3 weeks. Clinical evaluation confirmed the diagnosis of EN. Common infectious, inflammatory, and drug‐related causes were excluded. Due to a history of long‐standing constipation, serological testing for CD was performed, revealing markedly elevated antitissue transglutaminase IgA antibodies and positive antiendomysial IgA antibodies. CD was diagnosed according to ESPGHAN criteria, and a gluten‐free diet (GFD) was initiated. The EN lesions resolved within 3 weeks, which is compatible with the known self‐limiting course of the condition. We also reviewed previously published pediatric cases describing the co‐occurrence of EN and CD. Most reported patients exhibited additional clinical features suggestive of CD, including gastrointestinal symptoms, growth impairment, anemia, arthralgia, asthenia, or a previously diagnosed CD with recent dietary nonadherence. Although a temporal association between initiation of a GFD and resolution of EN has been described, a causal relationship cannot be established based on the available evidence.

## 1. Introduction

Celiac disease (CD) is a systemic, lifelong autoimmune disorder characterized by a complex interplay of genetic, environmental, and immunological factors. Its pathogenesis involves specific human leukocyte antigen (HLA) alleles—HLA‐DQ2 and HLA‐DQ8, exposure to dietary gluten, and the autoimmune response against the autoantigen tissue transglutaminase. Clinical presentation of CD is highly variable. Patients may exhibit gastrointestinal symptoms, extraintestinal manifestations, or remain asymptomatic [1]. Dermatological manifestations of CD can include dermatitis herpetiformis, psoriasis, alopecia areata, hereditary angioedema, atopic dermatitis, and chronic urticaria [2, 3].

Erythema nodosum (EN), the most prevalent form of panniculitis in children, manifests as tender, erythematous nodules primarily on the extensor surfaces of the lower extremities [4]. EN is generally considered a hypersensitivity reaction to diverse antigenic stimuli. While idiopathic in approximately 50% of the cases, EN may be associated with a variety of underlying conditions, including infections (e.g., streptococcal pharyngitis, and tuberculosis), sarcoidosis, inflammatory bowel disease (IBD), autoimmune diseases, medications, vaccinations, and malignancies [5].

In this report, we describe a child who initially presented with EN and was subsequently diagnosed with CD. In addition to presenting this case, we also review and analyze previously published pediatric reports describing the co‐occurrence of these two disorders, in order to better understand their potential association.

## 2. Case Presentation

A 2.5‐year‐old girl was referred for consultation with a 3‐week history of nodular lesions localized to the lower legs. The eruption was not accompanied by any systemic symptoms. According to the parents, since the age of one, the child had experienced infrequent bowel movements—twice per week—characterized by hard stools and straining, occasionally associated with pain. She was not receiving any regular medications, had no known allergies, and her vaccination schedule was up to date. There was no recent history of respiratory or gastrointestinal infections. Family history was unremarkable.

**FIGURE 1:**
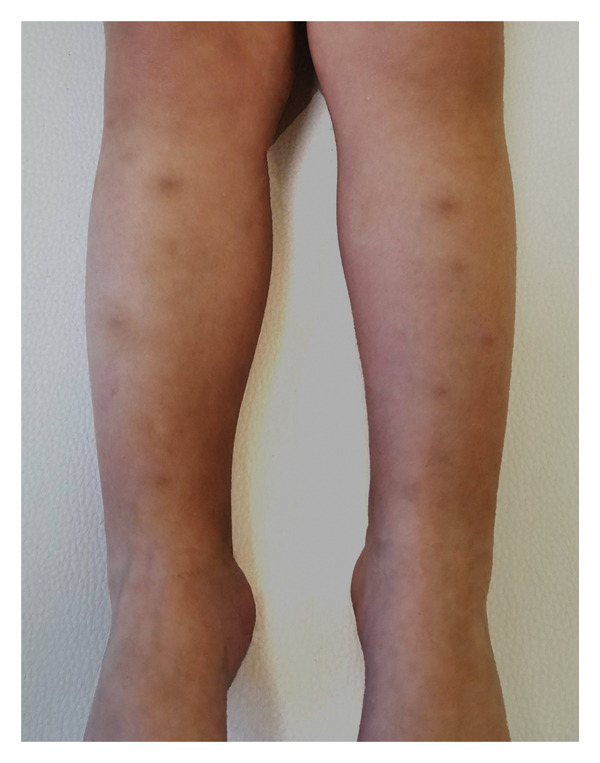
Pretibial nodules characteristic of erythema nodosum.

Physical examination revealed multiple erythematous to violaceous nodular lesions distributed over the pretibial areas of both lower legs (Figure 1). Anthropometric measurements were within normal limits: weight was 12.9 kg (42nd percentile), height 90 cm (39th percentile), and body mass index (BMI) 15.9 kg/m^2^ (49th percentile). The child appeared well, afebrile, and hemodynamically stable. No other abnormalities were noted.

Based on clinical appearance, a diagnosis of EN was made, and an extensive workup was initiated. Initial laboratory investigations revealed a normal erythrocyte sedimentation rate (5 mm/h) and C‐reactive protein (< 1 mg/L). Additional testing—including complete blood count, serum electrolytes, creatinine, liver enzymes, lactate dehydrogenase, alkaline phosphatase, antistreptolysin‐O titer, protein electrophoresis, albumin, immunoglobulins, and urinalysis—yielded normal results. A throat swab culture showed normal flora. Chest radiograph was unremarkable, with no signs of hilar or mediastinal lymphadenopathy. Abdominal ultrasound showed no pathological findings.

Given the history of persistent constipation, serological testing for CD was performed on an exploratory basis. Serum total IgA levels were within the normal range, while antitissue transglutaminase IgA antibodies (TTG‐IgA) were markedly elevated at 129 U/mL (normal < 10 U/mL). A second serum sample tested positive for antiendomysium IgA antibodies (EMA‐IgA) at a titer of 1:320. Based on the diagnostic criteria of the European Society for Paediatric Gastroenterology, Hepatology, and Nutrition (ESPGHAN) [6], a diagnosis of CD was established, and a gluten‐free diet (GFD) was initiated. Three weeks after the initiation of the GFD, the skin lesions had resolved and stool consistency had returned to normal. In accordance with ESPGHAN recommendations, both parents were tested for TTG‐IgA, and their results were within normal limits.

## 3. Discussion

While both CD and EN are individually well described in pediatric populations, reports of their co‐occurrence remain rare and primarily limited to isolated case reports. The nature of this potential association remains unclear. To further explore this relationship, we reviewed all available pediatric cases describing the concurrent presentation of CD and EN, summarizing the key clinical characteristics in Table 1.

**TABLE 1:** Summary of reported pediatric cases with concurrent celiac disease and erythema nodosum.

Author, year (ref.)	Age (yrs)	Sex	EN duration	Clinical and laboratory findings	Diagnosis	Resolution after GFD
Durand et al., 1991 [7]	17	F	6 month	Diarrhea, megaloblastic anemia	AGA‐IgA, duodenal biopsy	Not specified
Bartyik et al., 2004 [8]	16	F	∼4 yr (recurrent, then persistent)	Low serum iron	TTG‐IgA, EMA‐IgA, HLA typing, duodenal biopsy	1 mo
Fretzayas et al., 2011 [9]	10	M	2 month	Growth impairment	TTG‐IgA, HLA typing, duodenal biopsy	2 mo
Vaz et al., 2017 [10]	10	M	2‐3 d	CD with recent gluten exposure	TTG‐IgA and TTG‐IgG	2–4 wk^∗^
Abu‐Rumeileh et al., 2023 [11]	6	M	Not specified	Fever, headache, asthenia, lameness	Serology, duodenal biopsy	Not specified

*Note:* GFD, gluten‐free diet; AGA‐IgA, antigliadin IgA antibodies; EMA‐IgA, antiendomysial IgA antibodies; TTG‐IgA, antitissue transglutaminase IgA antibodies; TTG‐IgG, antitissue transglutaminase IgG antibodies.

Abbreviations: CD, celiac disease; EN, erythema nodosum; GFD, gluten‐free diet.

^∗^As described as “few weeks” in the original.

While our findings, along with previously published reports, point to a possible association between EN and CD, the available evidence is limited and should be interpreted with caution. This co‐occurrence raises several unresolved questions, including the role of HLA‐related genetic susceptibility, the potential contribution of CD‐related immune mechanisms to EN, whether a GFD contributes to symptom improvement, and the appropriateness of CD screening in selected pediatric patients with EN.

The immunogenetic background of EN is incompletely understood. While CD is strongly associated with specific Class II HLA alleles, particularly HLA‐DQ2 and HLA‐DQ8, no consistent HLA pattern has been established for EN [12]. Earlier studies suggested a possible association between EN and the Class I allele HLA‐B8; however, these findings have not been consistently replicated [13, 14]. Recent research indicates that HLA‐DRB1 allele frequencies may differ between idiopathic and secondary EN: idiopathic EN has been associated with a lower frequency of HLA‐DRB1∗04, whereas secondary EN, particularly in sarcoidosis, shows a higher frequency of DRB1∗13 [15]. Additionally, in patients with IBD and EN, a weak association has been observed with HLA‐B∗15 and the −1031 TNF‐α promoter polymorphism [16]. These observations suggest that genetic susceptibility in EN is heterogeneous and likely depends on the underlying etiology, rather than reflecting a single shared immunogenetic pathway.

It has been hypothesized that the loss of intestinal barrier integrity and immune activation in CD could contribute to systemic inflammatory manifestations through T‐lymphocyte activation and cytokine release following gluten exposure [17]. Although the mechanisms are not yet fully understood, the clinical improvement observed in certain autoimmune conditions following the initiation of a GFD may be partly due to the cessation of this immune activation and the subsequent reduction in cytokine‐mediated inflammation [18]. However, there is currently no direct evidence that these mechanisms contribute to the pathogenesis of EN, and any proposed immunopathogenetic association with CD should be regarded as hypothetical.

EN is a self‐limiting condition that typically resolves spontaneously within 2–8 weeks without specific treatment [19]; however, prolonged or recurrent forms may occur in the presence of a persistent antigenic or inflammatory stimulus [8]. Among the previously reported pediatric cases, the duration of EN symptoms prior to diagnosis varied considerably, ranging from a few days to over 1 year [7–10]. In cases with available follow‐up, resolution of EN was observed within 2–8 weeks after the initiation of a GFD [8–10]. In our patient, EN resolved 3 weeks after starting the diet, following a total disease duration of 6 weeks. Considering the self‐limiting nature of EN, the small number of reported cases, and the presence of incomplete data, it remains unclear whether the resolution observed after the initiation of a GFD reflected the natural course of the condition or was influenced by the intervention.

In light of these uncertainties, the question arises whether routine screening for CD should be recommended in all children presenting with EN. Although universal screening for CD in children with EN has been proposed by some authors [4, 9], others recommend limiting the diagnostic workup to recurrent or persistent cases [8, 10, 20], particularly when the etiology remains unclear [21]. The reported cases describing the association between EN and CD involved patients who exhibited additional symptoms or signs suggestive of CD, such as diarrhea, growth impairment, low serum iron, megaloblastic anemia, arthralgia, asthenia, or a known diagnosis of CD with recent dietary noncompliance [7–11]. In our case, the child had long‐standing constipation, which may also be considered a gastrointestinal manifestation of CD. Nevertheless, the current evidence is insufficient to support universal serological screening for CD in children with isolated EN. Any diagnostic evaluation should, therefore, be individualized, performed on an exploratory basis, and guided by accompanying clinical features or known risk factors.

## 4. Conclusion

The co‐occurrence of EN and CD in children is rare and has primarily been documented in isolated case reports. While temporal associations have been described, including in the present case, the available evidence does not support a causal relationship. EN and CD are, therefore, more likely to represent two coexisting conditions rather than EN being directly caused by CD. Evaluation for CD in children with EN may be considered on an exploratory basis when additional gastrointestinal symptoms or extraintestinal features suggestive of CD are present.

## Author Contributions

Momcilo Pavlovic: contributed to the conceptualization of the work, wrote the original draft, participated in the critical review and editing of the manuscript, and provided overall supervision.

Karolina Berenji: contributed to the original draft preparation, critical review and editing of the manuscript, supervision of the writing process, and conducted the literature search and review.

Zeljko Rokvic: participated in the review and editing of the manuscript and was involved in the clinical assessment and diagnostic evaluation of the patient.

## Funding

The authors received no specific funding for this work.

## Disclosure

All authors have read and approved the final version of the manuscript and agreed to be accountable for all aspects of the work.

## Ethics Statement

Ethical approval was not required for this case report, in accordance with institutional guidelines.

## Consent

Although no identifiable personal information is disclosed in this report, written informed consent was obtained from the patient’s parents for publication of this case report and any accompanying images.

## Conflicts of Interest

The authors declare no conflicts of interest.

## Data Availability

Data used to support the findings of this study are available on request from the corresponding author.
